# Association Between Non-traditional Lipid Indices and Sarcopenia: Evidence from a Prospective Chinese Cohort Study

**DOI:** 10.1007/s11596-026-00193-w

**Published:** 2026-04-23

**Authors:** Rui-rong Pan, Rui-xue Tang, Zi-fan Qian, Jun-yan Gong

**Affiliations:** Department of Geriatrics, Affiliated Hospital of Jiangsu University, Zhenjiang, 212001 China

**Keywords:** Sarcopenia, Lipid metabolism, Biomarkers, Frailty, China Health and Retirement Longitudinal Study (CHARLS), Lipoprotein combined index (LCI), Non-high-density lipoprotein cholesterol to high-density lipoprotein cholesterol ratio (NHHR)

## Abstract

**Objective:**

Recent study links sarcopenia with lipid metabolism disorders. This study aimed to examine the associations between non-traditional lipid indices—specifically the lipoprotein combined index (LCI) and the non-high-density lipoprotein cholesterol to high-density lipoprotein cholesterol ratio (non-HDL-C to HDL-C ratio, NHHR)—and incident sarcopenia in a Chinese population.

**Methods:**

Participants aged ≥ 45 years were enrolled from the 2011–2015 waves of the China Health and Retirement Longitudinal Study (CHARLS). Multivariate logistic regression was applied in cross-sectional analyses (n = 3,161) to assess the associations between log-transformed LCI, NHHR and sarcopenia. Longitudinal analyses (n = 2,898) were performed using multivariate Cox proportional hazards models, restricted cubic splines, subgroup analyses, and receiver operating characteristic (ROC) curves. Sensitivity analyses were conducted to test the robustness of the results.

**Results:**

Higher quartiles of lnLCI and lnNHHR were inversely associated with sarcopenia in both cross-sectional (lnLCI Q4: OR 0.58, *P* = 0.015; lnNHHR Q3: OR 0.56, *P* = 0.005) and longitudinal analyses (lnLCI Q4: HR 0.57, *P* = 0.001; lnNHHR Q4: HR 0.46, *P* = 0.002), with linear trends observed in spline models. Alcohol intake modified the association between lnNHHR and sarcopenia (*P* = 0.017), whereas the association of lnLCI with sarcopenia remained consistent in alcohol intake subgroup. ROC analyses showed similar predictive ability (lnLCI AUC 0.599; lnNHHR AUC 0.603). Sensitivity analyses excluding participants with hypercholesterolemia further validated the findings.

**Conclusions:**

Among Chinese adults aged ≥ 45 years, LCI and NHHR were inversely associated with sarcopenia, with NHHR demonstrating slightly superior diagnostic potential.

**Supplementary Information:**

The online version contains supplementary material available at 10.1007/s11596-026-00193-w.

## Introduction

Sarcopenia is an age-associated condition marked by the gradual decline of skeletal muscle mass and strength. While this condition manifests primarily in older populations, it may also present in middle-aged individuals and has emerged as a major public health concern [1]. Sarcopenia demonstrates robust associations with diverse negative clinical consequences, including fractures, cognitive decline, and metabolic disorders, compared to individuals without this condition [2]. A global epidemiological study has demonstrated that sarcopenia significantly impacts the quality of life for 10%–16% of the elderly population worldwide [3]. Its prevalence is disproportionately higher among individuals with comorbid conditions, notably cancer [4], liver cirrhosis [5], renal dysfunction [6], and metabolic disorders [7]. In the Chinese elderly population, the prevalence of sarcopenia reaches 20.7% [8], substantially exceeding rates documented in other Asian populations, such as Japan (9.9% [9]) and South Korea (13.01% [10]). Moreover, sarcopenic patients incur a heavier healthcare burden [11]. These findings underscore the pressing necessity for efficient early detection and preventative measures for sarcopenia.

Clinical instruments for evaluating sarcopenia differ significantly, with each possessing intrinsic limitations. For example, bio-electrical impedance analysis (BIA) and functional tests can be influenced by physical factors such as age, sex, hydration status, comorbidity, joint problems, and neurological deficits [12, 13]. As the current diagnostic criteria for sarcopenia are linked to the onset or progression of the disease, they are not suitable for early diagnosis and targeted intervention to prevent sarcopenia [14]. Therefore, it is critical to explore reliable potential biomarkers for this condition. Existing research has established a close association between sarcopenia development and chronic low-grade inflammation [15]. Moreover, intramyocellular and interstitial deposition of lipid species contributes to lipotoxicity, further exacerbating inflammatory cascades [16]. This pathological link provides a solid rationale for exploring lipid-related parameters as potential biomarkers for sarcopenia. The association between dyslipidaemia and sarcopenia has been further substantiated by a recent meta-analysis, which reported correlations with commonly measured lipid parameters (i.e., high-density lipoprotein cholesterol [HDL-C], low-density lipoprotein cholesterol [LDL-C], and the triglyceride to high-density lipoprotein cholesterol [TG/HDL-C] ratio), although the nature and underlying mechanisms of these correlations remain incompletely elucidated [17]. However, a single lipid parameter may be insufficient to adequately characterize the overall physiological status of the organism, whereas composite lipid indices may offer superior discriminatory and predictive performance. For instance, in predicting in-hospital mortality among patients with acute coronary syndrome, traditional lipid markers demonstrate limited prognostic value, while nontraditional composite lipid indices show a stronger correlation with adverse clinical outcomes [18].

Emerging as non-traditional lipid biomarkers, the lipoprotein combined index (LCI) and non-HDL-C to HDL-C ratio (NHHR) represent novel metabolic indicators. Relevant studies have validated their capacity to predict the risk of multiple chronic pathologies, including cardiovascular disease, stroke, diabetes, and obstructive sleep apnoea (OSA) [19–23]. Although a US-based study documented a positive association between NHHR and sarcopenia, its external validity is constrained by distinct geodemographic characteristics [24]. Furthermore, no research to date has investigated the potential association between LCI and sarcopenia.

Considering the above research gaps, this study utilized data from the China Health and Retirement Longitudinal Study (CHARLS) to explore the association between non-traditional lipid indices (specifically LCI and NHHR) and the risk of incident sarcopenia among middle-aged and older Chinese individuals, and further evaluated their predictive and diagnostic value for sarcopenia.

## Materials and Methods

### Study Methodology and Population

We analyzed prospective data derived from the China Health and Retirement Longitudinal Study (CHARLS), a nationally representative cohort of community-dwelling Chinese adults aged 45 years and older. The 2011 baseline survey enrolled a total of 17,705 participants across 150 prefecture-level cities or districts in 28 Chinese provinces.

**Fig. 1:**
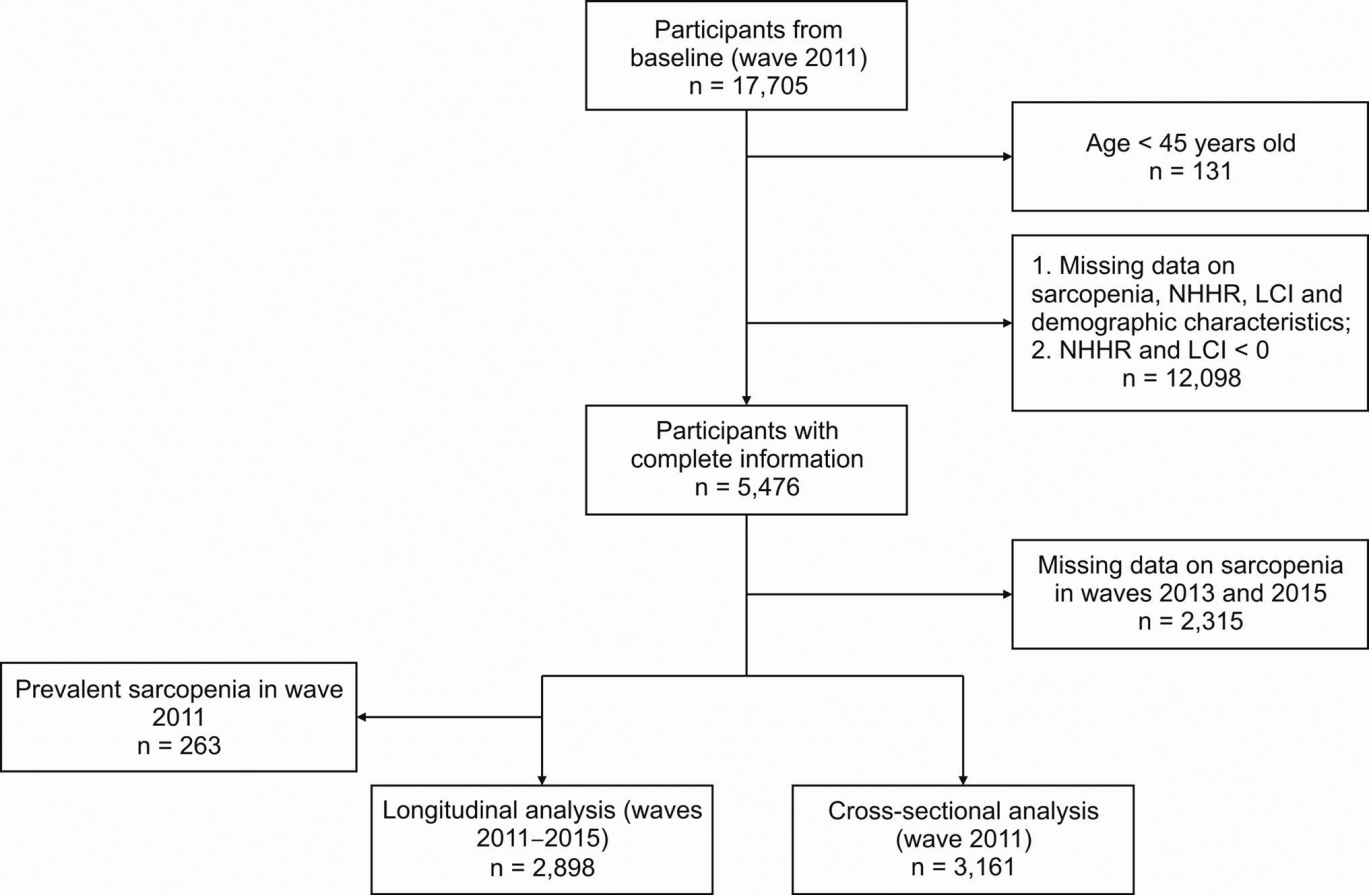
Flow chart of participant enrolment and exclusion in the cross-sectional and longitudinal analyses

For the present study, data were primarily extracted from the 2011, 2013, and 2015 survey waves. Key biological markers, including venous blood samples, were collected in the 2011 and 2015 waves, while anthropometric measurements (i.e., height, weight, and handgrip strength) were assessed at all three time points. Consistent with typical longitudinal cohort designs, gradual participant attrition was observed across follow-up periods, including the 2013 follow-up wave. Participants were excluded sequentially based on the following prespecified criteria: (1) missing 2011 baseline data for anthropometric, demographic, lifestyle, lipid profile, or chronic disease-related variables (n = 12,097); (2) missing sarcopenia-related assessment data in either the 2013 or 2015 wave (n = 2,315); (3) age younger than 45 years at baseline (n = 131); (4) biologically implausible values for the NHHR or LCI values less than 0 (n = 1); and (5) for the longitudinal analysis specifically, prevalent sarcopenia identified at the 2011 baseline (n = 263). The final analytical samples comprised 3,161 participants for the cross-sectional analysis (2011 wave) and 2,898 participants for the longitudinal analysis (2011–2015 waves) (Fig. 1).

### Assessment of Sarcopenia

Sarcopenia was defined in accordance with the 2019 criteria from the Asian Working Group for Sarcopenia (AWGS 2019), which requires concurrent reduced handgrip strength (men < 28 kg; women < 18 kg) and low appendicular skeletal muscle mass index (ASMI) (men < 7.0 kg/m^2^; women < 5.4 kg/m^2^) [25]. Appendicular skeletal muscle mass (ASM) was estimated using a validated regression formula specific to Chinese adults, as follows: ASM = 0.193 × weight (kg) + 0.107 × height (cm) − 4.157 × sex (1 = men, 2 = women) − 0.037 × age (years) − 2.63121 [26]. This formula demonstrates excellent agreement with dual-energy X-ray absorptiometry (DXA) and is widely applied in large-scale population-based epidemiological studies [27, 28]. The ASMI was subsequently calculated by normalizing ASM to the square of height, using the formula: ASMI = ASM/height^2^.

### Assessment of LCI and NHHR

The LCI was calculated using the following formula: LCI = (total cholesterol [TC] × triglycerides [TG] × LDL-C)/HDL-C [23]. The NHHR was defined as: NHHR = non-HDL-C/HDL-C [24], where non-HDL-C was computed as the difference between TC and HDL-C.

### Statistical Analysis

Data normality was assessed via the Kolmogorov–Smirnov test. Continuous variables were presented as means with standard deviations (SD) and medians with interquartile ranges (IQR) based on distribution characteristics, while categorical variables were expressed as proportions. Given the non-normal distribution of raw LCI and NHHR values, both indices were subjected to natural logarithmic transformation to approximate a Gaussian distribution. The logarithmically transformed variables (lnLCI, lnNHHR) were subsequently categorized into four quartile groups (Q1–Q4), with the lowest quartile (Q1) set as the reference group for subsequent analyses.

Multivariate logistic regression models were applied to examine the cross-sectional associations between quartiles of lnLCI/lnNHHR and baseline sarcopenia (2011), with three sequentially adjusted models constructed: Model 1 was unadjusted; Model 2 was adjusted for age and sex; Model 3 was the fully adjusted model, controlling for a comprehensive set of covariates including age, sex, body mass index (BMI), waist circumference (WC), ASM, alcohol consumption, smoking status, educational attainment, marital status, residential region, and physician-diagnosed chronic diseases. Cox proportional hazards regression models were used to explore the longitudinal associations between baseline lnLCI/lnNHHR and incident sarcopenia during follow-up, with identical covariate adjustments as the fully adjusted logistic model. Restricted cubic spline (RCS) regression with three knots was performed to detect potential non-linear correlations between the lipid indices and sarcopenia risk. Subgroup analyses coupled with interaction tests were conducted to evaluate potential effect heterogeneity across different participant subgroups.

Receiver operating characteristic (ROC) curve analysis was carried out to assess the diagnostic performance of lnLCI and lnNHHR for identifying prevalent sarcopenia. Optimal cutoff values were determined using the Youden index, calculated as sensitivity plus specificity minus one. To test the robustness of the primary findings, sensitivity analyses were performed by excluding participants with hypercholesterolemia to minimize potential confounding effects of this condition. All statistical analyses were performed using R software (version 4.3.3; R Foundation for Statistical Computing) via RStudio, and a two-sided *P* value < 0.05 was considered statistically significant.

## Results

### Features of the Population

The cross-sectional analysis included 3,161 baseline participants (263 with sarcopenia and 2,898 without sarcopenia), who were categorized into quartiles based on lnLCI and lnNHHR levels. The longitudinal analysis comprised 2,898 participants (145 with incident sarcopenia and 2,753 without incident sarcopenia), with identical quartile stratification applied for both lipid indices (Table 1).

**Table 1:** Features of the population

**Characteristics**	**Cross-sectional study**				**Longitudinal study**			
	Overall	Without sarcopenia	With sarcopenia	*P* value	Overall	Without sarcopenia	With sarcopenia	*P* value
n	3,161	2,898	263		2,898	2,753	145	
Age, median [IQR]	59.0 [53.0, 67.0]	58.0 [52.0, 65.0]	71.0 [64.0, 77.0]	< 0.001	58.0 [52.0, 65.0]	57.0 [51.0, 64.0]	67.0 [61.0, 74.0]	< 0.001
Age, n (%)				< 0.001				< 0.001
45–59 years	1,675 (53.0)	1,639 (56.6)	36 (13.7)		1,639 (56.6)	1,607 (58.4)	32 (22.1)	
60–75 years	1,256 (39.7)	1,111 (38.3)	145 (55.1)		1,111 (38.3)	1,024 (37.2)	87 (60.0)	
> 75 years	230 (7.3)	148 (5.1)	82 (31.2)		148 (5.1)	122 (4.4)	26 (17.9)	
Gender, n (%)				0.537				0.838
Female	1,614 (51.1)	1,485 (51.2)	129 (49.0)		1,485 (51.2)	1,409 (51.2)	76 (52.4)	
Male	1,547 (48.9)	1,413 (48.8)	134 (51.0)		1,413 (48.8)	1,344 (48.8)	69 (47.6)	
Height (cm), median [IQR]	157.8 [152.1, 164.2]	158.1 [152.5, 164.8]	153.5 [147.7, 158.4]	< 0.001	158.1 [152.5, 164.8]	158.4 [152.9, 165.0]	154.1 [147.5, 160.0]	< 0.001
Weight (kg), median [IQR]	57.9 [51.0, 65.7]	58.8 [52.0, 66.3]	49.5 [43.8, 54.4]	< 0.001	58.8 [52.0, 66.3]	59.1 [52.3, 66.6]	52.5 [45.7, 56.8]	< 0.001
WC (cm), median [IQR]	84.5 [77.8, 92.0]	85.0 [78.0, 92.1]	79.8 [73.1, 86.0]	< 0.001	85.0 [78.0, 92.1]	85.1 [78.0, 92.2]	80.0 [75.2, 87.3]	< 0.001
BMI (kg/m^2^), median [IQR]	23.1 [20.9, 25.7]	23.3 [21.1, 25.9]	20.7 [18.9, 22.6]	< 0.001	23.3 [21.1, 25.9]	23.4 [21.2, 26.0]	21.6 [19.9, 23.8]	< 0.001
Max Grip Strength (kg), median [IQR]	32.0 [25.0, 40.0]	33.0 [26.5, 40.0]	17.2 [14.9, 24.2]	< 0.001	33.0 [26.5, 40.0]	33.0 [27.0, 40.0]	28.5 [22.5, 34.0]	< 0.001
TC (mg/dL), median [IQR]	190.2 [167.8, 214.9]	189.8 [167.8, 214.9]	192.5 [168.6, 214.2]	0.845	189.8 [167.8, 214.9]	190.2 [167.8, 214.6]	188.3 [165.1, 217.7]	0.689
TG (mg/dL), median [IQR]	104.4 [75.2, 148.7]	105.3 [75.2, 150.4]	89.4 [67.3, 131.9]	< 0.001	105.3 [75.2, 150.4]	106.2 [76.1, 152.2]	85.0 [67.3, 127.4]	< 0.001
LDL (mg/dL), median [IQR]	114.0 [93.9, 137.2]	114.0 [94.3, 136.9]	116.4 [93.4, 138.2]	0.572	114.0 [94.3, 136.9]	114.0 [94.3, 136.9]	114.0 [91.2, 132.2]	0.468
HDL-C (mg/dL), median [IQR]	49.5 [40.6, 60.7]	49.1 [40.2, 60.3]	53.7 [45.0, 64.4]	< 0.001	49.1 [40.2, 60.3]	49.1 [39.8, 59.9]	54.5 [45.6, 68.0]	< 0.001
ASM (kg), median [IQR]	15.0 [12.0, 18.0]	15.0 [12.0, 18.0]	12.0 [9.0, 14.0]	< 0.001	15.0 [12.0, 18.0]	15.0 [12.0, 18.0]	13.0 [10.0, 15.0]	< 0.001
ASMI (kg/m^2^), median [IQR]	6.0 [5.0, 7.0]	6.0 [5.0, 7.0]	5.0 [4.0, 6.0]	< 0.001	6.0 [5.0, 7.0]	6.0 [5.0, 7.0]	5.0 [4.0, 6.0]	< 0.001
LCI, median [IQR]	45,868.6 [25,281.2, 83,788.1]	46,459.5 [25,724.8, 85,682.9]	38,207.5 [20,523.7, 70,274.3]	< 0.001	46,459.5 [25,724.8, 85,682.9]	47,336.8 [26,116.3, 87,115.3]	33,152.8 [21,919.7, 61,392.9]	< 0.001
lnLCI, mean (SD)	10.7 (0.9)	10.8 (0.9)	10.5 (0.9)	< 0.001	10.8 (0.9)	10.8 (0.9)	10.5 (0.9)	< 0.001
NHHR, median [IQR]	2.8 [2.1, 3.7]	2.8 [2.1, 3.7]	2.5 [1.8, 3.5]	< 0.001	2.8 [2.1, 3.7]	2.8 [2.1, 3.8]	2.4 [1.8, 3.2]	< 0.001
lnNHHR, mean (SD)	1.0 (0.4)	1.0 (0.4)	0.9 (0.4)	< 0.001	1.0 (0.4)	1.0 (0.4)	0.9 (0.5)	< 0.001
Education, n (%)				< 0.001				< 0.001
Illiterate	831 (26.3)	704 (24.3)	127 (48.3)		704 (24.3)	644 (23.4)	60 (41.4)	
Elementary school	1,349 (42.7)	1,241 (42.8)	108 (41.1)		1,241 (42.8)	1,176 (42.7)	65 (44.8)	
Middle school and above	981 (31.0)	953 (32.9)	28 (10.6)		953 (32.9)	933 (33.9)	20 (13.8)	
Marriage, n (%)				< 0.001				0.007
Divorced	35 (1.1)	33 (1.1)	2 (0.8)		33 (1.1)	33 (1.2)	0 (0.0)	
Married	2,509 (79.4)	2,362 (81.5)	147 (55.9)		2,362 (81.5)	2,257 (82.0)	105 (72.4)	
Never married	32 (1.0)	26 (0.9)	6 (2.3)		26 (0.9)	25 (0.9)	1 (0.7)	
Separated	29 (0.9)	23 (0.8)	6 (2.3)		23 (0.8)	22 (0.8)	1 (0.7)	
Widowed	556 (17.6)	454 (15.7)	102 (38.8)		454 (15.7)	416 (15.1)	38 (26.2)	
Living area, n (%)				0.004				0.001
Rural	2,590 (81.9)	2,357 (81.3)	233 (88.6)		2,357 (81.3)	2,223 (80.7)	134 (92.4)	
Urban	571 (18.1)	541 (18.7)	30 (11.4)		541 (18.7)	530 (19.3)	11 (7.6)	
Smoking, n (%)				0.337				0.13
No	1,861 (58.9)	1,714 (59.1)	147 (55.9)		1,714 (59.1)	1,619 (58.8)	95 (65.5)	
Yes	1,300 (41.1)	1,184 (40.9)	116 (44.1)		1,184 (40.9)	1,134 (41.2)	50 (34.5)	
Drinking, n (%)				0.158				0.08
Drinking less than once a month	272 (8.6)	257 (8.9)	15 (5.7)		257 (8.9)	248 (9.0)	9 (6.2)	
Drinking more than once a month	771 (24.4)	710 (24.5)	61 (23.2)		710 (24.5)	683 (24.8)	27 (18.6)	
No drinking	2,118 (67.0)	1,931 (66.6)	187 (71.1)		1,931 (66.6)	1,822 (66.2)	109 (75.2)	
Hypertension, n (%)				0.266				0.698
No	2,356 (74.5)	2,168 (74.8)	188 (71.5)		2,168 (74.8)	2,062 (74.9)	106 (73.1)	
Yes	805 (25.5)	730 (25.2)	75 (28.5)		730 (25.2)	691 (25.1)	39 (26.9)	
Diabetes, n (%)				0.143				0.296
No	2,999 (94.9)	2,755 (95.1)	244 (92.8)		2,755 (95.1)	2,614 (95.0)	141 (97.2)	
Yes	162 (5.1)	143 (4.9)	19 (7.2)		143 (4.9)	139 (5.0)	4 (2.8)	
Heart diseases, n (%)				0.522				0.363
No	2,785 (88.1)	2,557 (88.2)	228 (86.7)		2,557 (88.2)	2,433 (88.4)	124 (85.5)	
Yes	376 (11.9)	341 (11.8)	35 (13.3)		341 (11.8)	320 (11.6)	21 (14.5)	
Lung diseases, n (%)				< 0.001				0.015
No	2,801 (88.6)	2,586 (89.2)	215 (81.7)		2,586 (89.2)	2,466 (89.6)	120 (82.8)	
Yes	360 (11.4)	312 (10.8)	48 (18.3)		312 (10.8)	287 (10.4)	25 (17.2)	
Stroke, n (%)				0.298				0.875
No	3,098 (98.0)	2,843 (98.1)	255 (97.0)		2,843 (98.1)	2,700 (98.1)	143 (98.6)	
Yes	63 (2.0)	55 (1.9)	8 (3.0)		55 (1.9)	53 (1.9)	2 (1.4)	
lnLCI, n (%)				0.002				< 0.001
Q1	799 (25.3)	710 (24.5)	89 (33.8)		710 (24.5)	655 (23.8)	55 (37.9)	
Q2	813 (25.7)	746 (25.7)	67 (25.5)		746 (25.7)	708 (25.7)	38 (26.2)	
Q3	808 (25.6)	743 (25.6)	65 (24.7)		743 (25.6)	712 (25.9)	31 (21.4)	
Q4	741 (23.4)	699 (24.1)	42 (16.0)		699 (24.1)	678 (24.6)	21 (14.5)	
lnNHHR, n (%)				< 0.001				< 0.001
Q1	802 (25.4)	710 (24.5)	92 (35.0)		710 (24.5)	653 (23.7)	57 (39.3)	
Q2	808 (25.6)	734 (25.3)	74 (28.1)		734 (25.3)	704 (25.6)	30 (20.7)	
Q3	805 (25.5)	757 (26.1)	48 (18.3)		757 (26.1)	717 (26.0)	40 (27.6)	
Q4	746 (23.6)	697 (24.1)	49 (18.6)		697 (24.1)	679 (24.7)	18 (12.4)	

*WC* waist circumference, *BMI* body mass index, *ASM* appendicular skeletal muscle mass, *ASMI* appendicular skeletal muscle mass index, *LCI* lipoprotein combined index, *NHHR* non-HDL-C/HDL-C ratio, *HDL-C* high-density lipoprotein cholesterol, *LDL-C* low-density lipoprotein cholesterol, *TC* total cholesterol, *TG* triglycerides

In both study cohorts, participants with sarcopenia were significantly older than those without sarcopenia (median age: 71 vs. 58 years in the cross-sectional cohort; 67 vs. 57 years in the longitudinal cohort; both *P* < 0.001), and a larger proportion of them were aged 60–75 years (55.1% vs. 38.3% in the cross-sectional group; 60.0% vs. 37.2% in the longitudinal group). Across both cohorts, individuals with sarcopenia consistently showed lower values for a series of anthropometric and biochemical parameters (all *P* < 0.001), including height, weight, WC, BMI, maximum handgrip strength, ASM, ASMI, TG, LCI, lnLCI, NHHR, and lnNHHR. In contrast, HDL-C levels were significantly higher in the sarcopenia group (53.7 mg/dL vs. 49.1 mg/dL in the cross-sectional analysis; 54.5 mg/dL vs. 49.1 mg/dL in the longitudinal analysis; both *P* < 0.001).

Significant between-group differences were also observed in sociodemographic characteristics. Participants with sarcopenia had lower educational attainment and higher marriage rates (both *P* < 0.001), a smaller proportion lived in urban areas (11.4% vs. 18.7% in the cross-sectional group; 7.6% vs. 19.3% in the longitudinal group; both *P* < 0.05), and chronic lung disease was more prevalent in this group (18.3% vs. 10.8% in the cross-sectional group; 17.2% vs. 10.4% in the longitudinal group; both *P* < 0.05). No significant between-group differences were detected for TC, LDL-C, sex, smoking status, alcohol consumption, hypertension, diabetes mellitus, or cardiovascular disease.

### Analysis of lnLCI and lnNHHR Levels with Sarcopenia Using Multivariate Logistic Regression

**Fig. 2:**
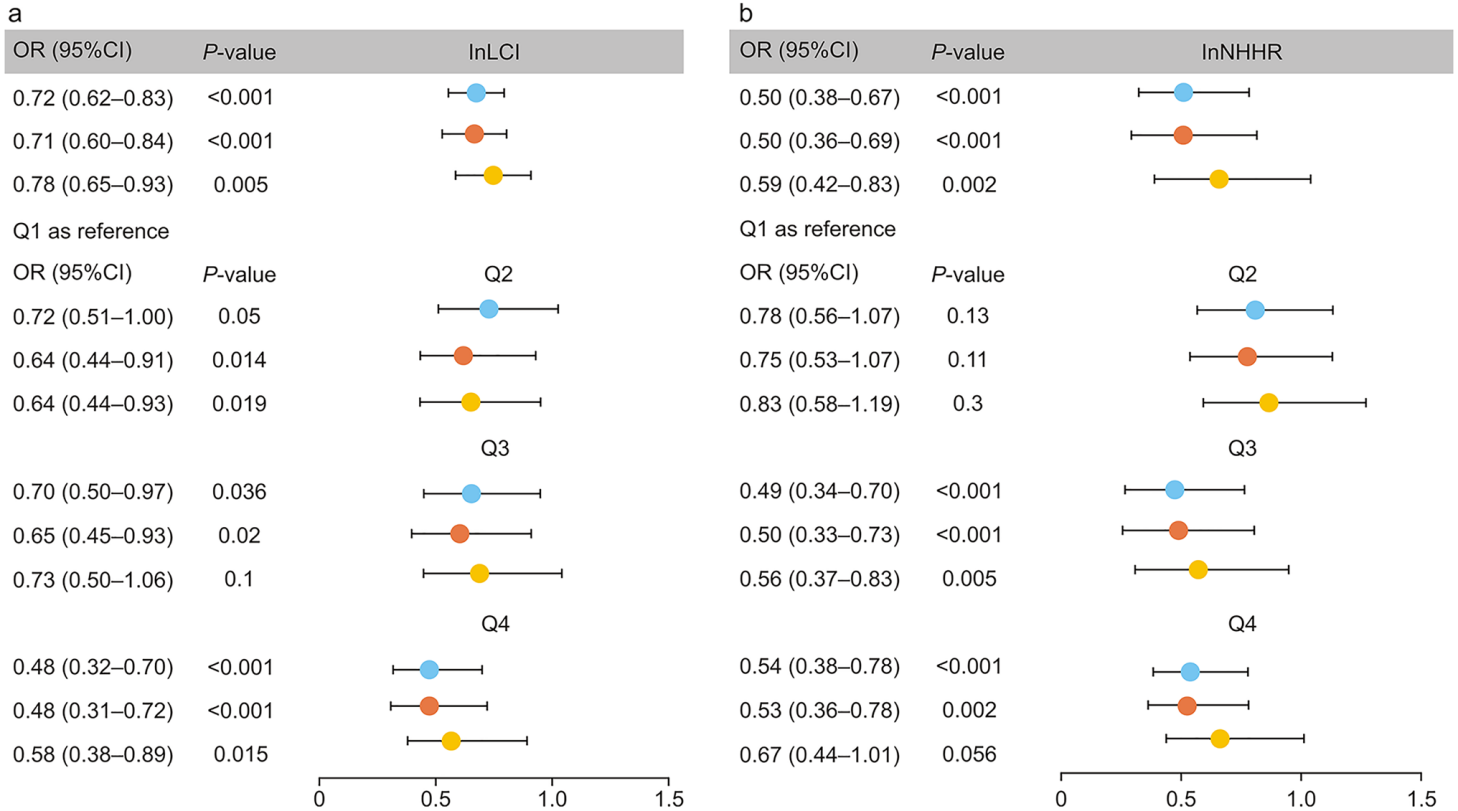
Multivariate logistic analysis of lnLCI (**a**) and lnNHHR (**b**) levels with sarcopenia in cross-sectional analyses. Model 1 (blue points): unadjusted; Model 2 (orange points): adjusted for sex and age; Model 3 (yellow points): further adjusted for age, sex, body mass index (BMI), waist circumference (WC), appendicular skeletal muscle mass (ASM), alcohol consumption, smoking status, education attainment, marital status, residential region, and physician-diagnosed diseases. OR, odds ratio; HR, hazard ratio; CI, confidence interval

Multivariable-adjusted logistic regression models were applied to evaluate the independent associations between lnLCI, lnNHHR and sarcopenia status (Fig. 2). Per 1-unit increment in lnLCI was associated with decreased odds of sarcopenia, with corresponding odds ratios (ORs) and 95% confidence intervals (CIs) as follows: Model 1 (unadjusted): OR = 0.72, 95% CI 0.62–0.83; Model 2 (age- and sex-adjusted): OR = 0.71, 95% CI 0.60–0.84; Model 3 (fully adjusted): OR = 0.78, 95% CI 0.65–0.93 (all *P* < 0.01). Quartile-based analysis revealed consistently reduced odds of sarcopenia in the highest lnLCI quartile (Q4) compared with the reference quartile (Q1), with OR values of 0.48 (95% CI 0.32–0.70), 0.48 (95% CI 0.31–0.72), and 0.58 (95% CI 0.38–0.89) across Models 1–3, respectively. Significant inverse associations were also detected in the second quartile (Q2) in Models 2 and 3 (Fig. 2a).

Similarly, per 1-unit increase in lnNHHR was linked to lower odds of sarcopenia: Model 1: OR = 0.50, 95% CI 0.38–0.67; Model 2: OR = 0.50, 95% CI 0.36–0.69; Model 3: OR = 0.59, 95% CI 0.42–0.83 (all *P* < 0.01). Quartile stratification analysis demonstrated consistent protective associations for the third quartile (Q3) relative to Q1 across all three models, with ORs ranging from 0.49 to 0.56 (all *P* < 0.01). The highest quartile (Q4) of lnNHHR was significantly protective in Models 1 and 2 (both *P* < 0.01), whereas this association attenuated to borderline significance in the fully adjusted Model 3 (OR = 0.67, 95% CI 0.44–1.01, *P* = 0.056). No significant associations between lnNHHR and sarcopenia risk were observed for Q2 across all adjusted models (Fig. 2b).

### Analysis of lnLCI and lnNHHR Levels with Sarcopenia Using Multivariate COX Regression

**Fig. 3:**
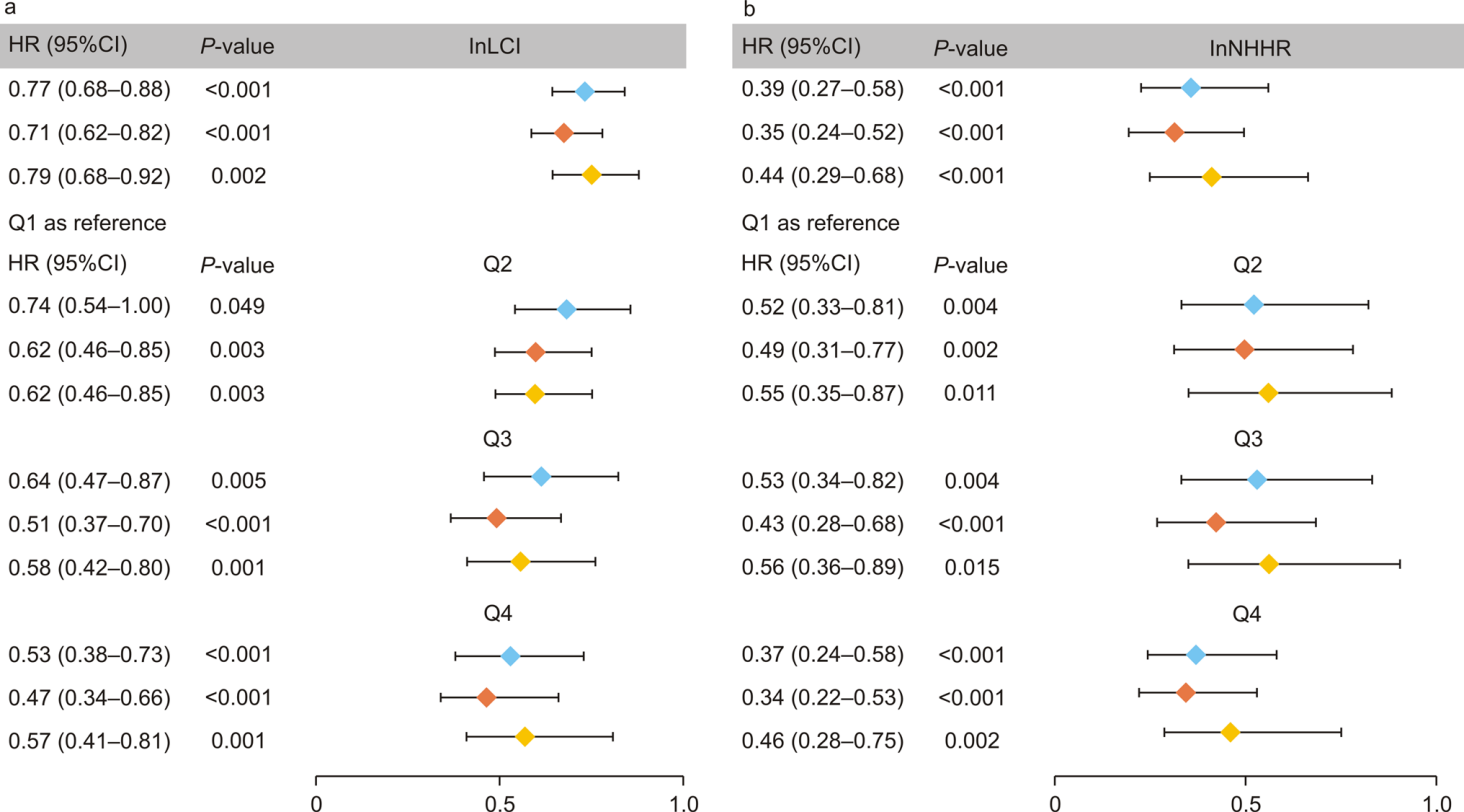
Multivariate COX regression analysis of lnLCI (**a**) and lnNHHR (**b**) levels with sarcopenia in longitudinal analyses. Model 1 (blue points): unadjusted; Model 2 (orange points): adjusted for sex and age; Model 3 (yellow points): further adjusted for age, sex, BMI, WC, ASM, alcohol consumption, smoking status, education attainment, marital status, residential region, and physician-diagnosed diseases

Cox proportional hazards regression models were conducted to further explore the longitudinal associations between lnLCI, lnNHHR and the risk of incident sarcopenia during follow-up. Both lipid indices were assessed as continuous variables and by quartile categorization, with results visualized in Fig. 3. Each unit increase in lnLCI was associated with HRs of 0.77 (95% CI 0.68–0.88; *P* < 0.001), 0.71 (95% CI 0.62–0.82; *P* < 0.001), and 0.79 (95% CI 0.68–0.92; *P* = 0.002) in Models 1–3, respectively.

Quartile-based analyses, with the lowest quartile (Q1) set as the reference group, revealed a progressive decline in HRs for incident sarcopenia from Q2 to Q4 across all sequential models, indicating a graded inverse association (Fig. 3a). Similarly, lnNHHR was inversely associated with incident sarcopenia risk when analyzed as a continuous variable across all three models. Quartile stratification of lnNHHR also demonstrated progressively lower HRs from Q2 to Q4 relative to the reference Q1 group, with all associations remaining statistically significant across unadjusted and adjusted models (Fig. 3b).

### Analysis of the Non-linear Association Through RCS

**Fig. 4:**
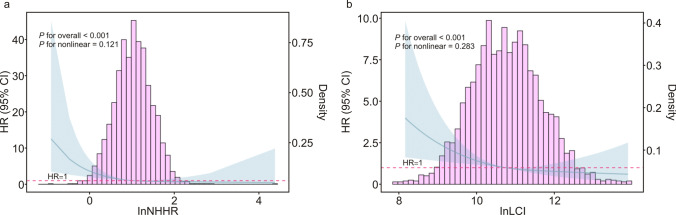
RCS curves analysis for the association between lnNHHR (**a**), lnLCI (**b**) levels and the risk of developing sarcopenia. The light purple dashed line was for HR = 1. The model was adjusted for age, sex, BMI, WC, ASM, alcohol consumption, smoking status, education attainment, marital status, residential region, and physician-diagnosed diseases

RCS regression with three knots was performed to examine potential non-linear associations between the lipid indices (LCI, NHHR) and the risk of sarcopenia (Fig. 4). Following full adjustment for covariates, both lnLCI and lnNHHR exhibited significant inverse relationships with sarcopenia risk (both *P* < 0.001). Importantly, no evidence of non-linearity was detected in the RCS analysis (*P* for non-linearity = 0.283 for lnLCI; *P* for non-linearity = 0.121 for lnNHHR), confirming a linear inverse relationship between these lipid indices and sarcopenia risk.

### Analysis of Subgroups on the Link between lnLCI, lnNHHR Levels, and Sarcopenia

**Fig. 5:**
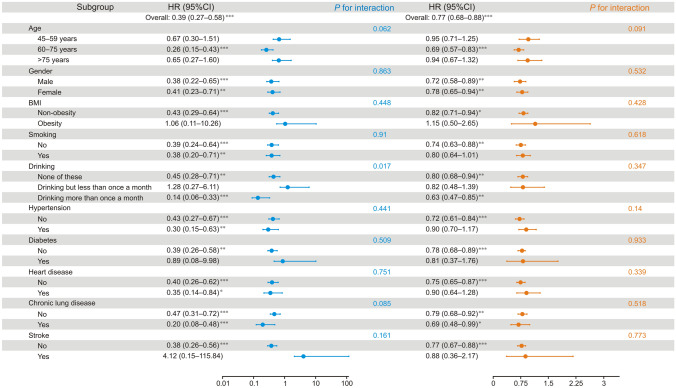
Subgroup analysis the association between lnLCI and lnNHHR levels and sarcopenia. Stratified analysis of the association between lnLCI (orange points and lines), lnNHHR (blue points and lines) and sarcopenia. When BMI is greater than or equal to 28 kg/m^2^, it was defined as obesity, otherwise it is classified as non-obesity. ^*^P < 0.05, ^**^P < 0.01, ^***^P < 0.001

Subgroup analyses were performed to evaluate the associations of LCI and NHHR with sarcopenia risk stratified by sex, age, BMI, lifestyle factors, and comorbidities, with interaction tests conducted (Fig. 5). In the overall study population, higher lnLCI was associated with a lower risk of incident sarcopenia (HR = 0.77, 95% CI 0.68–0.88, *P* < 0.001), with the most pronounced protective effect observed among participants aged 60–75 years (HR = 0.69, *P* < 0.001). No significant interactions were identified for age, sex, BMI, smoking status, alcohol consumption, or comorbidities, suggesting a consistent inverse association of lnLCI across subgroups.

Alcohol consumption significantly modified the association between lnNHHR and incident sarcopenia (*P*-interaction = 0.017). Stratified analyses revealed a stronger protective effect of lnNHHR among drinkers (HR = 0.14, 95% CI 0.06–0.33) compared with non-drinkers (HR = 0.45, 95% CI 0.28–0.71), indicating that drinking status may modify the protective association of NHHR with sarcopenia risk.

### ROC Curve Analysis for Evaluating the Diagnostic Performance

**Fig. 6:**
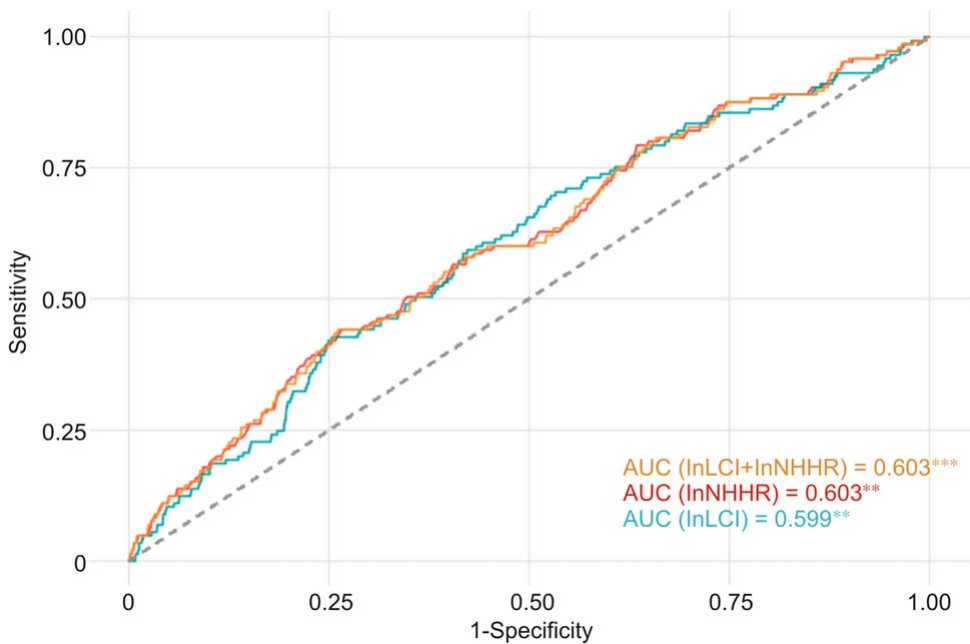
Comparison of ROC curves for diagnostic performance of lnLCI and lnNHHR in sarcopenia. ROC curves demonstrate diagnostic accuracy of lnNHHR (red solid line), lnLCI (blue solid line), and their combination (yellow solid line) in identifying sarcopenia. The diagonal dotted line represents the reference (AUC = 0.5). Analysis was based on the longitudinal cohort. ^*^P < 0.05, ^**^P < 0.01, ^***^P < 0.001

ROC curve analysis was conducted to assess the discriminatory capacity of LCI and NHHR for identifying prevalent sarcopenia (Fig. 6). The area under the curve (AUC) values were 0.599 (95% CI 0.550–0.650) for lnLCI and 0.603 (95% CI 0.548–0.642) for lnNHHR, respectively. Optimal cutoff values derived from the Youden index were 10.183 for lnLCI (specificity = 0.746, sensitivity = 0.427) and 0.772 for lnNHHR (specificity = 0.746, sensitivity = 0.427). Combined assessment of both lipid indices yielded comparable diagnostic performance (AUC = 0.603; 95% CI 0.555–0.651), with an optimal combined cutoff value of 0.245 (specificity = 0.738, sensitivity = 0.441).

### Sensitivity Analyses

Sensitivity analyses were conducted to verify the robustness of the primary findings. Participants with non-HDL-C ≥ 160 mg/dL [29], a threshold indicative of hypercholesterolemia, were excluded from the analytical sample to minimize potential confounding effects of abnormal lipid metabolism on the associations between LCI, NHHR, and sarcopenia risk.

Statistical models were adjusted for the identical set of covariates as employed in the main analyses, and the results were largely consistent with the primary findings (Table S1). When modelled as continuous variables, both lnLCI and lnNHHR remained significantly inversely associated with the risk of incident sarcopenia (lnLCI HR = 0.54, 95% CI 0.40–0.73, *P* < 0.001; lnNHHR: HR = 0.31, 95% CI 0.18–0.55, *P* < 0.001). Quartile-based analyses further corroborated this pattern: participants in the highest quartile of lnLCI (Q4: HR = 0.13, 95% CI 0.03–0.53, *P* = 0.005) and lnNHHR (Q4: HR = 0.44, 95% CI 0.22–0.88, *P* = 0.021) demonstrated markedly reduced sarcopenia risk compared with the reference quartile (Q1).

Collectively, these results indicate that the inverse associations between lnLCI, lnNHHR, and sarcopenia risk are not confounded by hypercholesterolemia, thereby reinforcing the validity and generalizability of our core findings. The consistent direction and magnitude of effects observed across sensitivity analyses support the potential of lnLCI and lnNHHR as robust protective biomarkers for sarcopenia in community-dwelling older Chinese adults.

## Discussion

Using data from the 2011, 2013, and 2015 CHARLS waves, we examined the associations of two non-traditional lipid indices—LCI and NHHR—with sarcopenia in cross-sectional and longitudinal analyses. Following log-transformation, both lnLCI and lnNHHR were inversely associated with sarcopenia after covariate adjustment. Alcohol intake modified the association between lnNHHR and sarcopenia, whereas the association of lnLCI with sarcopenia remained consistent. Both indices exhibited comparable predictive performance, and sensitivity analyses excluding participants with hypercholesterolemia confirmed the robustness of the findings.

Sarcopenia is increasingly recognized as an age-related condition, with muscle strength typically beginning to decline around the age of 40 [1, 30]. Besides advanced age, other risk factors for sarcopenia include underweight status, female sex, and the presence of chronic diseases [31]. These factors often interact and cumulatively accelerate muscle degeneration, thereby increasing the heterogeneity of disease onset and progression [32]. However, traditional diagnostic approaches rely heavily on the manifestation of overt symptoms, such as measurable declines in muscle strength or physical performance, which limits the effective identification of individuals at the early or preclinical stages and the timely implementation of preventive measures [14]. Consequently, investigating biomarkers that reflect underlying pathophysiological changes may further enhance early clinical detection and facilitate proactive intervention, potentially enabling risk stratification and individualized preventive strategies before irreversible functional decline occurs.

Sarcopenia arises from a complex interplay of interrelated mechanisms, including chronic inflammation, malnutrition, imbalanced protein turnover, oxidative stress, and mitochondrial dysfunction [33]. Rather than acting independently, these mechanisms interact synergistically, ultimately disrupting skeletal muscle homeostasis and accelerating age-related muscle degeneration. Concurrently, the development of sarcopenia can lead to disability, falls, osteoporosis, dyslipidemia, increased cardiovascular risk, metabolic syndrome, and immunosuppression [1, 34].

Less commonly emphasized, lipid intermediates such as fatty acids also play critical roles in modulating skeletal muscle quality and function. Beyond serving as energy substrates, lipid intermediates actively participate in cellular signalling pathways that regulate muscle metabolism and structural integrity [35]. The accumulation of lipids and their metabolites can occur both intracellularly within myocytes and in the surrounding interstitial tissue, contributing to lipotoxicity, inducing inflammation, and subsequently impairing muscle strength and function [16, 36]. Additionally, clinical evidence indicates that dyslipidemia is highly prevalent in patients with sarcopenia. For instance, chronic obstructive pulmonary disease (COPD) is significantly comorbid with sarcopenia, and over half of COPD patients are hypercholesterolemic [37]. Furthermore, a study of patients with ankylosing spondylitis reported a significant reduction in muscle mass among male patients, accompanied by a high prevalence of dyslipidemia in this population [38]. These findings suggest that dyslipidemia may not only be a consequence of sarcopenia but also a potential driving factor in its onset and progression.

Chronic low-grade inflammation, also referred to as “inflammaging,” is widely regarded as a central mechanistic link connecting dyslipidemia and sarcopenia [39]. Chronic inflammation plays a pivotal role in sarcopenia, involving signalling pathways associated with protein metabolism, obesity, lipid infiltration, and immunosenescence [35, 40–42]. Prior studies have demonstrated that inflammatory markers such as tumor necrosis factor-α (TNF-α), C-reactive protein (CRP), interleukin-6 (IL-6), and interleukin-1β (IL-1β) increase with age and are directly correlated with the loss of muscle mass and strength [42–44]. These pro-inflammatory cytokines can impair muscle protein synthesis, enhance proteolytic pathways, and exacerbate mitochondrial dysfunction, thereby contributing to progressive declines in muscle function [45, 46]. Dyslipidemia, particularly hypercholesterolemia, can further exacerbate inflammatory responses. A study has shown that in diabetic myopathy, elevated hyperlipidemia aggravates inflammation-induced pyroptosis, resulting in significant muscle loss, sarcopenia, and adverse skeletal muscle remodeling [47].

In addition, inflammatory markers have been shown to modulate the association between specific lipid ratios and the risk of sarcopenia, including the NHHR, LDL-C/HDL-C ratio, and remnant cholesterol (RC)/HDL-C ratio [48]. In a cross-sectional study by Zhao et al., LCI was found to correlate with both dyslipidaemia and inflammation in obese individuals [49]. These findings suggest that dyslipidaemia may contribute to sarcopenia not only through metabolic dysregulation but also via inflammation-mediated pathways, highlighting a potential interaction between lipid metabolism and chronic low-grade inflammation [50].

It has long been established that elevated HDL-C levels are protective against cardiovascular disease [27]. However, emerging evidence indicates that high HDL-C levels may induce cellular senescence and act as pro-inflammatory factors, increasing the risk of sarcopenia in older Chinese populations [51, 52]. Conversely, mild increases in TG, TC, or LDL-C levels within the normal range may be protective against sarcopenia in this population [53]. A hospital-based case–control study demonstrated that lipoprotein ratios outperformed conventional lipid parameters in predicting coronary heart disease in a Chinese population [54]. Collectively, single lipid biomarkers are insufficient to comprehensively reflect the body’s lipid metabolic status. Composite lipid indices, by contrast, can provide more holistic information and may exhibit superior performance in disease prediction and risk assessment. For example, in a study of 2631 participants, Wang et al. found that the TG/HDL-C ratio had a protective effect against sarcopenia (OR: 0.63, 95% CI 0.49–0.81) [55]. Similarly, in a study of 7993 participants, Duan et al. reported an inverse relationship between the atherogenic index of plasma (AIP) and sarcopenia (HR: 0.73, 95% CI 0.62–0.86) [56], which is consistent with our findings. Our results indicate that these non-traditional lipid indices (LCI and NHHR), which reflect the musculoskeletal–endocrine interplay, are protective against sarcopenia in older adults, with low levels potentially serving as markers for identifying individuals at higher risk. Thus, incorporating these accessible lipid indicators into routine clinical screening could enable clinicians to identify high-risk populations earlier, thereby expanding the scope of preventive strategies and facilitating personalized recommendations.

In contrast to our findings, a U.S. study identified a positive association between NHHR and both sarcopenia and lower grip strength [24]. This discrepancy may be attributed to differences in sarcopenia diagnostic criteria, ethnic composition of the study populations, or more complex interaction mechanisms that require further investigation. These divergent results underscore the need for region-specific validation of lipid-based sarcopenia biomarkers, a gap that our study addresses. To the best of our knowledge, no prior research has investigated the association between LCI and sarcopenia, and our study is the first to fill this critical research void.

Despite its methodological strengths, including the prospective cohort design and comprehensive covariate adjustment, our study has several limitations. First, although we employed multivariable Cox and logistic regression models to adjust for numerous confounders, residual confounding cannot be entirely ruled out. Notably, smoking status, alcohol consumption, and medical history were self-reported, which may introduce misclassification or recall bias. Second, sarcopenia was defined using a formula-derived estimation of ASM rather than direct DXA measurement, although this formula-based method has been validated and exhibits high consistency with DXA results. Finally, our study was restricted to individuals aged ≥ 45 years, yet muscle loss may originate in early adulthood and is increasingly affecting younger populations [57], highlighting the need for future studies in younger cohorts.

In conclusion, our findings demonstrate that LCI and NHHR—cost-effective, routinely measurable lipid metabolism markers in clinical practice—may serve as valuable indicators for implementing early preventive strategies against sarcopenia. These indices offer opportunities for multidisciplinary prevention and management of sarcopenia in older adults. Critically, in healthcare settings where advanced diagnostic modalities are unavailable, LCI and NHHR provide an economically viable alternative for proactive screening of individuals at high risk of sarcopenia, thereby facilitating timely intervention and potentially improving clinical outcomes.

## Supplementary Information

Below is the link to the electronic supplementary material.

Supplementary file 1 (DOCX 21 KB)

## Data Availability

The datasets generated and analyzed during the current study are available in the China Health and Retirement Longitudinal Study (CHARLS) repository: https://charls.pku.edu.cn/.
