# Supplementary material for: Association Between Non-traditional Lipid Indices and Sarcopenia: Evidence from a Prospective Chinese Cohort Study
Source: Curr Med Sci. 2026 Apr 23;46(3):802–14. doi: 10.1007/s11596-026-00193-w (PMC13315467; doi:10.1007/s11596-026-00193-w)
Supplement: Supplementary file 1 — (DOCX 21 KB) [file 11596_2026_193_MOESM1_ESM.docx]

**Table. S1 Multivariate Cox regression analysis in non-hypercholesterolemia group**

| Characteristic | Model 1 | | | Model 2 | | | Model 3 | | |
| --- | --- | --- | --- | --- | --- | --- | --- | --- | --- |
|  | HR | 95% CI | *P*-value | HR | 95% CI | *P*-value | HR | 95% CI | *P*-value |
| lnLCI | 0.55 | 0.41, 0.72 | <0.001 | 0.47 | 0.35, 0.63 | <0.001 | 0.54 | 0.40, 0.73 | <0.001 |
| InLCI, Quartile |  |  |  |  |  |  |  |  |  |
| Q1 | As reference |  |  |  |  |  |  |  |  |
| Q2 | 0.66 | 0.43, 1.02 | 0.058 | 0.57 | 0.37, 0.88 | 0.011 | 0.57 | 0.37, 0.89 | 0.013 |
| Q3 | 0.49 | 0.30, 0.81 | 0.006 | 0.4 | 0.24, 0.67 | <0.001 | 0.48 | 0.28, 0.82 | 0.007 |
| Q4 | 0.11 | 0.03, 0.47 | 0.003 | 0.1 | 0.02, 0.40 | 0.001 | 0.13 | 0.03, 0.53 | 0.005 |
| lnNHHR | 0.31 | 0.18, 0.50 | <0.001 | 0.26 | 0.16, 0.43 | <0.001 | 0.31 | 0.18, 0.55 | <0.001 |
| InNHHR, Quartile |  |  |  |  |  |  |  |  |  |
| Q1 | As reference |  |  |  |  |  |  |  |  |
| Q2 | 0.55 | 0.35, 0.88 | 0.012 | 0.55 | 0.35, 0.88 | 0.012 | 0.62 | 0.38, 0.99 | 0.045 |
| Q3 | 0.41 | 0.23, 0.71 | 0.002 | 0.34 | 0.19, 0.60 | <0.001 | 0.45 | 0.25, 0.81 | 0.008 |
| Q4 | 0.38 | 0.20, 0.73 | 0.004 | 0.33 | 0.17, 0.63 | <0.001 | 0.44 | 0.22, 0.88 | 0.021 |
